# Barriers and facilitators to infection prevention and control in Dutch residential care facilities for people with intellectual and developmental disabilities: A theory-informed qualitative study

**DOI:** 10.1371/journal.pone.0258701

**Published:** 2021-10-29

**Authors:** Famke Houben, Mitch van Hensbergen, Casper D. J. Den Heijer, Nicole H. T. M. Dukers-Muijrers, Christian J. P. A. Hoebe

**Affiliations:** 1 Department of Sexual Health, Infectious Diseases and Environmental Health, South Limburg Public Health Service, Heerlen, The Netherlands; 2 Department of Social Medicine, Care and Public Health Research Institute (CAPHRI), Faculty of Health, Medicine and Life Sciences, Maastricht University, Maastricht, The Netherlands; 3 Department of Medical Microbiology, Care and Public Health Research Institute (CAPHRI), Faculty of Health, Medicine and Life Sciences, Maastricht University Medical Centre (MUMC+), Maastricht, The Netherlands; 4 Department of Health Promotion, Care and Public Health Research Institute (CAPHRI), Faculty of Health, Medicine and Life Sciences, Maastricht University, Maastricht, The Netherlands; Thomas Jefferson University, UNITED STATES

## Abstract

**Background:**

Care institutions are recognised to be a high-risk setting for the emergence and spread of infections and antimicrobial-resistant organisms, which stresses the importance of infection prevention and control (IPC). Accurate implementation is crucial for optimal IPC practice. Despite the wide promotion of IPC and research thereof in the hospital and nursing home setting, similar efforts are lacking in disability care settings. Therefore, this study aimed to assess perceived barriers and facilitators to IPC among professionals working at residential care facilities (RCFs) for people with intellectual and developmental disabilities (IDD), as well as to identify professional-reported recommendations to improve IPC.

**Methods:**

This qualitative study involved semi-structured interviews (before COVID-19) with twelve professionals from five Dutch RCFs for people with IDD. An integrated theoretical approach was used to inform data collection and analysis. Thematic analysis using inductive and deductive approaches was conducted. This study followed the COnsolidated criteria for REporting Qualitative research (COREQ) guidelines.

**Results:**

Our findings revealed barriers and facilitators at the guideline, client, professional, professional interaction, professional client interaction, client interaction, organisational, community, and societal level. Six main themes covering multiple barriers and facilitators were identified: (1) guidelines’ applicability to (work)setting; (2) professionals’ cognitions and attitude towards IPC (related to educational background); (3) organisational support and priority; (4) educational system; (5) time availability and staff capacity; and (6) task division and change coaches. The main professional-reported recommendations were the introduction of tailored and practical IPC guidelines, structural IPC education and training among all professionals, and client participation.

**Conclusions:**

To promote IPC, multifaceted and multilevel strategies should be implemented, with a preliminary need for improvements on the guideline, professional, and organisational level. Given the heterogeneous character, i.e., different professionals, clients and care needs, there is a need for a tailored approach to implement IPC and sustain it successfully in disability care. Our findings can inform future IPC practice improvements.

## Background

In an institutional care environment, the opportunities for the onset of healthcare-associated infections (HAIs) and transmission of infections and antimicrobial-resistant organisms are abundant [1, 2]. This transmission potential tends to have a significant impact on infection and mortality rates [3, 4]. A Dutch study in nursing homes (NHs) reported a HAIs prevalence of 6.7%, 7.6% and 7.6%, in 2007, 2008 and 2009, ranging from 0–32.4% between NHs [5]. A follow-up of this study revealed an average HAIs prevalence of 2.2% in 2010–2017, varying from 0–37% by NH [6]. Another study conducted in European long-term care facilities (LTCFs) indicated a HAIs prevalence of 3.4% in 2013 [7]. Concerning the prevalence of antimicrobial-resistant organisms, a Dutch study revealed that 18.2% of LTCF residents were colonised with one or more multidrug-resistant bacteria in 2015, with especially high *Extended-Spectrum Beta-Lactamase (ESBL)*-carriage rates [8]. Studies conducted in the United States reported even higher prevalences of antimicrobial-resistant organisms among LTCF residents, ranging from 30 to 50% in 2002–2011 [9].

Infection prevention and control (IPC) is designed to decrease transmission potential, thereby minimising the rate of preventable infections and the acquisition of antimicrobial resistance (AMR) [10]. IPC includes preventive measures such as hand hygiene, use of personal protective equipment (e.g., gloves, masks), sterilising medical instruments and disinfecting environmental surfaces. Care facilities should focus on effective IPC implementation, given the health risks associated with AMR and emerging infections [3, 10]. In case new infectious diseases emerge, such as COVID-19, the implementation of IPC becomes even more crucial [11]. Despite the increased attention to IPC in the NH and long-term care setting [12, 13], current efforts and studies mainly focus on hospitals [12] and NHs for the elderly [14]. To our knowledge, no prior studies regarding IPC implementation have been conducted in residential care facilities (RCFs) for people with intellectual and developmental disabilities (IDDs). Examples of disabilities cared for in these facilities are intellectual disabilities (formerly mental retardation), autism spectrum disorders and Down syndrome. In the Netherlands, an estimated 440,000 people have an intellectual and/or developmental disability [15]. Of these individuals, approximately 111,010 people reside in RCFs. Disability care is recognised to be a high-risk setting for the emergence and spread of contagious pathogens [16]. Disability care facilities provide care to various groups of vulnerable people. Antibiotics are regularly prescribed within these institutions, which indicates a significant potential of developing AMR [10]. Besides medical interventions, the behaviour of both professionals and clients can be a risk factor for the onset and transmission of infections and antimicrobial-resistant organisms [16].

Promoting IPC implementation in RCFs for people with IDD requires change of several actors (e.g., care workers, clients, and managers) and at several levels (e.g., professional, and organisational level) [17]. To successfully achieve behavioural and organisational change, one must identify barriers and facilitators to IPC implementation. The identification of barriers and facilitators is most effective when supported by theory [17, 18]. By conducting a theoretical analysis of the factors impeding or facilitating IPC implementation, it is possible to understand the relationship between these factors and the mechanisms by which they influence change [18]. This allows researchers and policymakers to create theoretically informed interventions to improve IPC in disability care settings. According to implementation science, an intervention is more likely to be successful when users are involved during the development [18]. The present theory-informed study aimed to assess perceived barriers and facilitators to IPC among professionals working at RCFs for people with IDD, as well as to identify professional-reported recommendations to improve IPC.

## Methods

### Ethics statement

Ethical approval to conduct the study was obtained from the Ethics Committee of the Faculty of Psychology and Neuroscience at Maastricht University (ERCPN 188_10_02_2018_S4). The study was in compliance with the Declaration of Helsinki. Written informed consent was obtained from participants before the interviews.

### Design

A qualitative study was performed involving semi-structured interviews. As we aimed to assess perceived factors facilitating or impeding IPC, a qualitative approach was chosen since this allows exploration of perceptions and encourages participants to share rich descriptions and in-depth information [19]. We used a qualitative descriptive design to provide a comprehensive description of the factors facilitating or impeding IPC in disability care [20]. This study embedded the assessment of perceived barriers and facilitators to IPC in implementation science theory. The COnsolidated criteria for REporting Qualitative research (COREQ) guidelines [21] were followed for data reporting (S1 Appendix).

### Theory

Successful implementation of new practices depends on behavioural and organisational change [17]. According to theories of Grol and Wensing [17, 22] and Flottorp et al. [23], barriers and incentives to change in healthcare practice should be examined at six different levels: the innovation itself (i.e., guideline level), the individual professional, the patient, the social (i.e., professional interaction), organisational, and external environment level (i.e., economic, and socio-political context). To achieve a broad understanding of factors that could hamper or facilitate on each level, various implementation science theories were reviewed since they include different relevant concepts that influence change. These theories show similarities but have slightly different focal points, include rather different concepts, or use somewhat different formulations. Some theories are more directed to the characteristics of the innovation (e.g., compatibility and procedural clarity), such as the Measurement Instrument for Determinants of Innovations checklist [24]. Other theories focus more on underlying individual motivations for behaviours, like the Attitude—Social norm—self Efficacy (ASE) model and Integrated Change (I-change) model which explain behaviour by linking attitude, social influence and self-efficacy with intention and behaviour [25, 26]. The Health Belief model also attempts to predict behaviour by focusing on individual beliefs like risk perception [27]. Yet, these more individual-oriented theories are subjected to some criticism since they assess cognitive determinants of behaviour and neglect non-conscious processes like habit [28, 29]. There are also more ‘system oriented’ theories that explain the complex associations between individual, social, and environmental factors by identifying dimensions including individual, interpersonal, organisational, community, and public policy, so called socio-ecological models [30]. All aforementioned theories include relevant concepts for the assessment of barriers and facilitators to change, therefore an integrated theoretical approach was adopted when conducting the study (i.e., to inform data collection and analysis).

### Participant selection

Participants were professionals working at RCFs for people with IDD in the Netherlands. Since the disability care sector is characterised by a variety of different professionals [16], we aimed to compile a sample in which participants from a broad range of professions were represented. In doing so, we intended to achieve a broad understanding of the perceptions and needs regarding IPC in this particular care setting. Participants were recruited by snowball sampling [31, 32]. Initial recruitment started by contacting a physician specialised in disability care of five disability care institutions in the southern part of the Netherlands (Limburg and Brabant). This contact person recruited participants within their respective organisation to take part in the interviews and provided contact details of potential participants. Professionals were approached to take part in the interview either by e-mail, telephone or during a face-to-face meeting, after having been provided with a description of the study. Invited professionals were asked to recruit future participants among their co-workers. When professionals were willing to participate, an interview was planned, and an informed consent form was signed. Up to two reminders were sent via e-mail or telephone to professionals who did not respond to earlier invitations. Participants were recruited until data saturation was achieved [33].

### Data collection

Semi-structured audio-recorded interviews were conducted between October 2019 and March 2020 (before COVID-19) with professionals at their workplace. MvH (PhD student) and MD (junior researcher) conducted the interviews. Both interviewers were trained and experienced in conducting interviews and qualitative research. There was no relationship established between the interviewers and participants prior the study. The interviewers introduced themselves as researchers and elaborated on the study aim before the interviews. Before and during the interviews, the confidentiality of data was emphasised to minimise the possibility of receiving socially desirable answers. The interviews were guided by a topic guide, consisting of 25 questions (S2 Appendix). The topic guide was developed by CdH (PhD and MD, physician specialised in infection disease control) and MvH, and informed by implementation science theories (as described in the ‘theory’ section); the themes in the topic guide reflected major concepts from the theories. To ensure applicability, the guide was piloted among five healthcare professionals prior to conducting the interviews, including key informants regarding IPC and a physician specialised in disability care. This resulted in no major revisions, only slight modifications regarding the order of questions. Moreover, preliminary findings were presented in a focus group consisting of 20 disability care professionals, which provided the opportunity for critical reflection and validating the topic guide. The preliminary findings reflected the experiences and perceptions of the focus group participants; therefore, no revisions were made to the topic guide. The main themes included in the topic guide were descriptive data of the professionals such as occupation, age, years of experience; their attitude and perceptions towards IPC; the role of IPC in their daily work; social influences regarding IPC; the role of IPC at the organisational level; and recommendations to improve IPC.

### Data analysis

The interviews were transcribed verbatim in Dutch by a professional transcription service. The twelve transcripts in MS Word documents were imported into ATLAS.ti 8.4.2 software for qualitative analysis. Thematic analysis [34] with inductive and deductive approaches [35] was used to analyse the data. This hybrid approach allowed the integration of implementation science theories (as described in the ‘theory’ section) into the process of deductive thematic analysis while simultaneously allowing the direct emergence of themes from the data using inductive coding. We employed a realist approach, considering the whole data set and reporting experiences, meanings, and the reality of participants [36]. Choosing a realist approach meant that we focused on the manifest rather than the latent content of the interviews. The coding process followed Braun and Clarke’s analytic method for thematic analysis [34]: (i) transcript reading and familiarisation of data; (ii) initial coding across entire dataset (i.e., codes representing a specific barrier or facilitator); (iii) coding data by assessing interesting patterns and developing final codes; (iv) synthesising codes into themes and subthemes and developing a thematic map; (v) reviewing themes and assessing their consistency across the entire dataset; and (vi) finalising themes and subthemes. The codes and themes were created from observed patterns in the interview data and theoretical understanding gained during the review of implementation science theories (as described in the ‘theory’ section). To structure the data, emerging themes were assigned to the levels on which they occurred, based on a synthesis of the theory of Flottorp et al. [23], Grol and Wensing [17, 22] and socio-ecological models [30]: guideline, patient (in our case client), professional, professional interaction, organisational, community (in our case the disability care sector) and societal level. S3 Appendix provides an example of the coding process. Data analysis was performed by two researchers (FH, PhD student; MD) independently. Disagreements between coders were discussed in the expert-group until consensus was reached. The coding process was in addition peer-reviewed by a third researcher (MvH) to enhance the quality of data analysis.

## Results

In total, 18 professionals from five disability care institutions were approached. Of the invited professionals, 12 (66%) participated in the study. Reasons for non-participation were due to time constraints. In total, ten women and two men participated in the study. Of which, six client-based professionals (social worker, nurse, physician) and six managerial professionals (quality assurance officer, supervisor, manager). Professionals were on average 48.9 years old (range 37–64 years). The interviews lasted 47 minutes on average (range 34–60 minutes). Data saturation [33] was confirmed to be reached after data analysis, since no new information concerning barriers, facilitators and recommendations emerged after the tenth interview.

### Categories and themes

Qualitative analysis of the data revealed barriers and facilitators at the guideline, client, professional, professional interaction, professional client interaction, client interaction, organisational, community, and societal level. This aligns with the levels identified from the theory of Grol and Wensing [17, 22], Flottorp et al. [23] and socio-ecological models [30], though adds two additional levels regarding the social context: professional client interaction and client interaction. Each level includes themes, these themes comprise a variety of barriers and facilitators. Six main themes covering multiple barriers and facilitators were identified: (1) guidelines’ applicability to (work)setting; (2) professionals’ cognitions and attitude towards IPC; (3) organisational support and priority; (4) educational system; (5) time availability and staff capacity; and (6) task division and change coaches. An overview of all identified themes per level, corresponding to specific barriers and facilitators, is provided in Table 1. To conceptualise our findings, we created an integrated theoretical framework.

**Table 1:** Barriers and facilitators to IPC implementation, perceived by professionals working at residential care facilities for people with intellectual and developmental disabilities (n = 12), depicted per level of the integrated theoretical framework (see Fig 1) and categorised by corresponding theme.

Level	Barriers	Facilitators
**Guideline level**	*Accessibility* • Poor comprehensibility of IPC guidelines for non-medically educated professionals (e.g., social workers) *Applicability to (work)setting* • Lack of compatibility/applicability: lack of IPC guidelines tailored to the disability care setting • Poor feasibility/practicality: IPC guidelines too lengthy, lack illustrations	*Accessibility* • Sufficient access to IPC guidelines *Applicability to (work)setting* • Practical IPC guidelines, including schemes and illustrations, and clear procedural descriptions (procedural clarity)
**Client level (as reported by the professional)**	*Nature of disability and associated behaviour* • Difficult to instruct and teach clients IPC measures • Non-compliance and defiant behaviour *Cognitions and attitude* • Lack of hygiene awareness and low risk perception (due to lack of understanding associated with intellectual and/or developmental disability [IDD]) *Diversity in client groups* • Heterogeneous group of clients leads to differences in IPC application: more attention is paid to IPC in groups of clients with severe IDDs compared to groups of clients with mild IDDs	*Cognitions and attitude* • Willingness to adhere to IPC measures • Showing interest in IPC
**Professional level**	*Cognitions and attitude* • Lack of awareness towards IPC • Low risk perception: belief that the client has a low risk of infection • Negative professional attitude • No interest in IPC • Laxness/laziness towards IPC implementation • Resistance/lack of willingness to implement IPC • Forgetting IPC implementation • Belief that IPC is not important *Knowledge and skills* • Lack of IPC knowledge *Intention and motivation* • Lack of motivation to implement IPC *Habits and routines* • Stuck in (old) habits *Diversity in types of professionals* • Heterogeneous group of professionals leads to differences in cognitions, attitudes, and knowledge regarding IPC: negative cognitions and attitude towards IPC, and a lack of IPC knowledge are generally more prevalent among non-medically educated professionals (e.g., social workers) compared to medically educated professionals.	*Cognitions and attitude* • Positive professional attitude towards IPC • Self-efficacy: sufficient belief/confidence in own ability to implement IPC • Belief in own ability to come up with solutions when IPC application is hindered by a client’s behaviour *Knowledge and skills* • Sufficient IPC knowledge *Intention and motivation* • Intention and preparation (to action): undertaking actions and preparing to implement IPC *Habits and routines* • Implementation IPC is habit/part of routine
**Professional interaction**	*Feedback and monitoring* • Lack of feedback between professionals on IPC performance *Role models* • Lack of exemplary professional behaviour regarding IPC	*Feedback and monitoring* • Mutual feedback and accountability: Feedback between professionals (including supervisors), in which they address each other on improper IPC behaviour • Monitoring of IPC application between professionals *Collaboration* • Multidisciplinary collaboration • Informational collaboration
**Professional client interaction**	*Role models* • Lack of exemplary behaviour of professional towards client regarding IPC	*Feedback and monitoring* • Both professionals and clients monitor IPC application and hold each other accountable (feedback and accountability) *Social support* • Support and stimulation regarding IPC application from professional to client
**Client interaction**	*Peer influence* • Negative peer influence due to negative role models	-- ^a^
**Organisational level**	*Organisational support and priority* • Lack of structural organisational attention towards IPC • Lack of support board of directors and management • Lack of priority for IPC *Educational system* • Lack of structural IPC education and training among all staff *Time availability and staff capacity* • High work pressure • High staff turnover • Staff shortages *Task division and change coaches* • Lack of professionals responsible for IPC (e.g., infection control professional) *Leadership and institutional policy* • Lack of IPC policy • Lack of management involvement • Lack of enforcement in case of non-adherence to IPC *Resources and materials* • Lack of adequate IPC materials/equipment • Lack of financial resources	*Organisational support and priority* • Sense of urgency and organisational awareness towards the importance of IPC *Educational system* • Structural IPC education and training aimed at: • New employees • Non-medical educated professionals Clients *Task division and change coaches* • Professionals responsible for IPC (i.e., infection control professional, infection control committee) • Professionals acting as driving forces for IPC implementation *Leadership and institutional policy* • Preparedness: outbreak measures in place *Resources and materials* • Sufficient IPC materials, both educational materials (e.g., posters) as well as equipment (e.g., hand sanitisers) • Sufficient financial resources
**Community level (i.e., disability care sector)**	*Care sector-related social norms and culture* • Sectoral norm/culture in which emphasis is placed on domesticity and guidance (i.e., behavioural aspects), and fewer focus on medical aspects (i.e., IPC)^b^ *Interorganisational networks* • Lack of sectoral collaboration (i.e., collaboration between disability care facilities), no common good in the sector	*Care sector-related social norms and culture* • Sectoral shift in which IPC is regarded as collective concern *Interorganisational networks* • Interorganisational collaboration ○ Collaboration between disability care facilities and external health organisations (i.e., hospitals or public health services). ○ Collaboration between disability care facilities Sectoral events and meetings
**Societal level**	*Workforce* • Shortage of workforce	*Involvement governmental agencies and cues to action* • Sufficient information provision from governmental organisations • Visit of the health inspectorate • Governmental initiatives (e.g., projects) directed to IPC

^a^ No facilitators were reported for the level ‘client interaction’.

^b^ Mostly reported by medically educated professionals (e.g., nurses, physicians).

Abbreviation: IPC infection prevention and control.

Note. Concepts in italics are the themes which categorised the perceived barriers and facilitators.

### Integrated theoretical framework for factors influencing IPC in disability care settings

**Fig 1:**
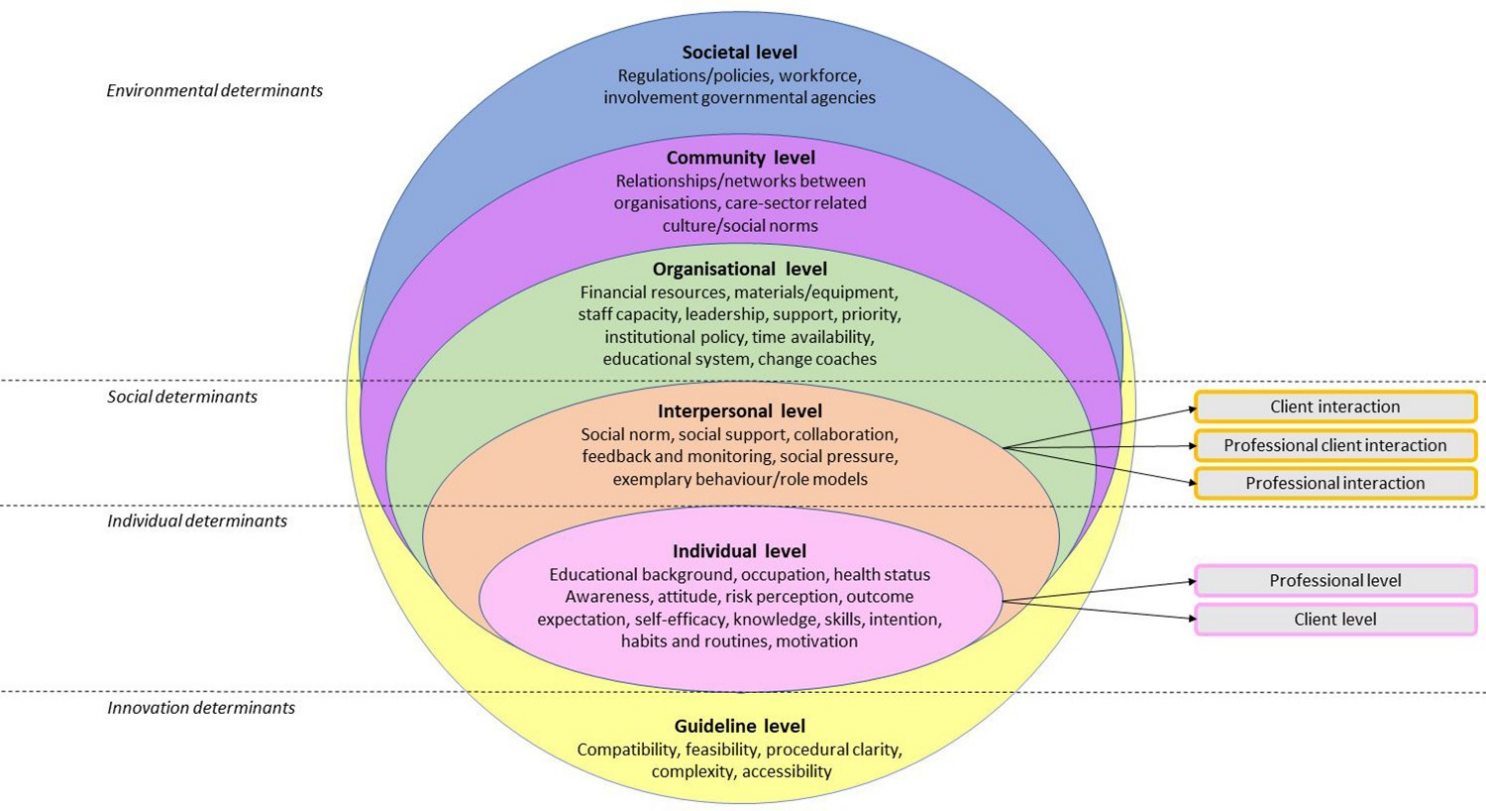
The integrated theoretical framework for factors influencing IPC in disability care settings, informed by various implementation science theories [17, 22–30], adapted to the results of our qualitative analysis. The integrated theoretical framework includes the guideline (yellow), individual (pink), interpersonal (orange), organisational (green), community (purple) and societal level (blue). The individual level comprises the client and professional level. The interpersonal level includes professional interaction, professional client interaction, and client interaction. The division of levels is based on the theories of Grol and Wensing [17, 22], Flottorp et al. [23] and socio-ecological models [30]. The interview data revealed that on the social context next to professional interaction [17, 22], professional client interaction and client interaction were important levels on which barriers and facilitators may occur. The underlying concepts of every level are based on various implementation science theories [17, 22–30], adapted to the data from our qualitative analysis.

We recognised strong parallels between our emergent findings and the concepts and levels identified in the relevant implementation science theories [17, 22–30]. Therefore, we synthesised these theories and adapted them to our results of the qualitative analysis to underpin our data mapping and reporting. This means that our framework evolved as the study went on (i.e., during data analysis) into the model that best describes our findings within the integrated implementation science theories [17, 22–30]. We propose an integrated theoretical framework for factors influencing IPC in disability care settings (Fig 1).

### Perceived barriers and facilitators to IPC

Perceived barriers and facilitators will be discussed per level and theme on which they occurred. An overview of all barriers and facilitators is provided in Table 1, displayed per level, and categorised by corresponding theme. Quotations were used to illustrate the findings, chosen based on the addition of contextual depth and richness these quotations bring to the narrative text.

#### Guideline level

*Accessibility*. Most participants indicated that guidelines are sufficiently available via a digital environment and thereby easily accessible for employees. Still, several professionals acknowledged that guidelines may lack accessibility to non-medically educated professionals due to difficulties with comprehensibility: “*If you do not have a nursing background*, *it is difficult to comprehend medical terms*. *Many employees are trained as social workers*, *not as nurses*. *We should simplify the language*.*” (P4*, *woman*, *40-45y nurse)*.

*Applicability to (work) setting*. A major theme was the applicability of existing guidelines to the work setting and disability care setting in general. The majority of participants reported guidelines lack practicality/feasibility and are often aimed at the NH or hospital setting: *“A lot of protocols are very detailed*, *long and policy-based*. *It needs to be simplified so that people can see all information in one glance*. *Also*, *a lot is focused on nursing homes*. *I would like to know how to deal with protocols and at the same time ensure a domestic environment*? *Without generalising the entire disability care sector*.” *(P12*, *woman*, *40-45y*, *quality assurance officer)*. Nevertheless, several institutions installed practical and user-friendly guidelines in which clear procedural descriptions are provided for every actor, which is perceived as facilitating: *“Our previous norovirus protocol turned out to be too difficult in practice*, *everyone was like*: *who should do what*? *Now we developed something which is easier to use and makes sure everyone knows what to do*.*” (P3*, *woman*, *35-40y*, *physician)*.

#### Client level

*Nature of clients’ disability and associated behaviour*. Participants recognised the difficulty of instructing and teaching clients IPC measures: *“The problem is often you cannot teach clients certain behaviour which results in complex situations*. *In terms of behaviour*, *they often do not want to*, *leading to major escalations*.*” (P7*, *woman*, *60-65y*, *nurse)*. Most participants perceived non-compliance and defiant behaviour among clients as important challenging factor: *“A client does not always accept*, *e*.*g*., *when I ask clients to put on clean clothes*, *there will be a lot of refusal*. *Eventually*, *they change into new clothes but when we leave*, *they put the dirty clothes back on*. *Or clients who say they are going to take a shower*. *They go into the bathroom*, *turn on the shower*, *make shower noises and wet their heads and say they showered*, *without actually showering*.*” (P5*, *man*, *35-40y*, *social worker)*.

*Clients’ cognitions and attitude*. Most participants acknowledged clients are often not aware of the infection risk and the importance of IPC, which is attributed to the lack of understanding due to a client’s intellectual ability: “*There is no understanding of hygiene anyway*. *Clients do not understand they can get sick from faeces*.” *(P8*, *woman*, *40-45y*, *supervisor)*. Nevertheless, several participants reported clients are in the end willing to comply to IPC measures: *“Practice shows that if we tell and educate them on why it is important or bring it with a little joke*, *everyone will always do the things we ask*.*” (P5*, *man*, *35-40y*, *social worker)*. Few participants noted that some clients show interest in IPC: *“Clients find IPC very interesting and are very enthusiastic about hygiene classes*. *Clients expressed they wanted to know more about hygiene and asked the nurse for a lesson on hygiene*.*” (P9*, *woman*, *45-50y*, *supervisor)*.

*Diversity in client groups*. IPC application differs per client group. Most participants reported that in general more attention is paid to IPC in groups with higher care needs (i.e., clients with severe IDD). Especially nurses perceived this as an important barrier, since in their regard clients with a mild intellectual disability are a major risk group: *“For clients who are more dependent on care*, *professionals control a large part of hygiene protocols*. *Whereas clients who are less care-dependent often take care of themselves*, *which poses a greater problem because hygiene is often missed*.*” (P4*, *woman*, *40-45y*, *nurse)*.

#### Professional level

*Professionals’ cognitions and attitude*. A key theme was a professional’s cognitions and attitude towards IPC. The most often reported professional-related barrier is the lack of awareness towards IPC: *“Care workers are often not aware and do not pay attention to IPC*, *because if you have a challenging group of clients*, *it is a real survival at work*.*” (P6*, *woman*, *60-65y*, *nurse)*. Other hindering factors are low risk perception and a negative professional attitude towards IPC: *“Professionals still go from sick clients to non-sick clients without protective equipment*. *The reason is laziness*, *a lack of interest or underestimation of the risk*. *Also*, *people dislike top-down orders*, *not everything is received with open arms*.*” (P4*, *woman*, *40-45y*, *nurse)*. Yet, the presence of a positive professional attitude is perceived to be vital for IPC implementation: “*To enable IPC*, *a change of attitudes is needed*.*” (P4*, *woman*, *40-45y*, *nurse)*. Another facilitator mentioned by several participants is self-efficacy. In particular, the belief in one’s own ability to come up with solutions even if the application of IPC measures is hindered by a client’s behaviour.

*Professionals’ knowledge and skills*. Several participants indicated the lack of IPC knowledge and skills as important barrier: *“Many people have no knowledge*, *nor do they have that background*. *They lack medical knowledge*, *that is an important issue*.*” (P12*, *woman*, *35-40y*, *quality assurance officer)*.

*Diversity in types of professionals*. The aforementioned barriers regarding cognitions, attitudes and knowledge were mainly reported by medically educated professionals. They recognised that a lack of awareness, low risk perception, negative attitude, and lack of knowledge regarding IPC are generally more prevalent among non-medically educated professionals: *“People who do not have a nursing background have no idea*. *Nurses are much more aware of this than social workers*.*” (P7*, *woman*, *60-65y*, *nurse)*.

*Professionals’ intention and motivation*. Half of the participants indicated their preparation (to action) and intention to implement IPC, which is perceived to positively influence actual application: *“I often have a pair of gloves in my back pocket*. *Especially since clients need affection and closeness*, *I make sure I have everything with me*.*” (P8*, *woman*, *40-45y*, *supervisor)*. One respondent perceived lack of motivation among professionals as the most important barrier: *“The biggest problem is motivation*. *With prevention you never immediately notice results*. *So*, *lots of people doubt about the added value*.*” (P10*, *man*, *55-60y*, *manager)*.

*Professionals’ habits and routines*. A few participants moreover reported that IPC is part of their routine, which is perceived as a facilitator: *“Many IPC measures became a routine for me*, *e*.*g*., *when I am going to wash someone*, *I put on gloves*. *I do not consciously think about it unless I notice it cannot be performed*.*” (P5*, *man*, *35-40y*, *social worker)*. Nonetheless, existing routines may also impede IPC in case professionals are stuck in old habits: *“People just do what they are used to*. *Those habits are ingrained*, *you are not aware of what you are doing and that things can be improved*.*” (P3*, *woman*, *35-40y*, *physician)*. One respondent perceived lack of motivation as the most important barrier: *“The biggest problem is motivation*. *With prevention you never immediately notice results*. *So*, *lots of people doubt about the added value*.*” (P10*, *man*, *55-60y*, *manager)*.

#### Professional interaction

*Feedback and monitoring between professionals*. More than half of the participants reported they address each other on professional behaviour in case IPC is not adequately applied. Feedback from supervisors is in particular perceived as a facilitator: *“If someone would not wear gloves*, *it would immediately be the topic of the day*. *We would hold each other to account*. *Our supervisor also calls on people regarding this*, *which is important*.*” (P5*, *man*, *35-40y*, *social worker)*. In line, professionals monitoring each other’s IPC behaviour is perceived as facilitating: “*We have to pay close attention to each other*, *e*.*g*., *check with your colleague whether it is performed properly*.” *(P2*, *woman*, *age unknown*, *nurse)*. At the same time, participants acknowledged that providing and receiving professional feedback is challenging: *“Recently*, *a colleague approached another colleague*, *who became angry and immediately called in sick the next day*.*” (P6*, *woman*, *60-65y*, *nurse)*.

*Professional role models*. Another barrier related to professional interaction is the lack of exemplary professional behaviour, which is especially perceived as a barrier when relating to supervisors: *“If the supervisor has fake nails*, *what is exemplary behaviour*? *What should she say to employees*?*” (P1*, *woman*, *55-60y manager)*.

*Collaboration*. Several participants indicated collaboration, especially multidisciplinary collaboration, as a stimulating factor: *“The moment we suspect an infection*, *cooperation with our medical service is fantastic*: *I can knock on their door and ask everything*. *If it is serious*, *they visit us immediately and everyone works together closely*: *the supervisor*, *care coordinator*, *medical service*, *as well as the remedial educationalist*, *which is very facilitating*.*” (P8*, *woman*, *40-45y*, *supervisor)*. Correspondingly, informational collaboration is regarded as a facilitator: *“When I doubt about something or I cannot find the guidelines*, *I ask a colleague*. *Also*, *oftentimes our physician provides us information about the guidelines or where to find them*.*” (P7*, *woman*, *60-65y*, *nurse)*.

#### Professional client interaction

*Social support from professional to client*. Several participants reported that the presence of a relationship based on trust and social support from professionals to clients positively affects IPC: *“Clients especially need contact and affection*. *If you have trust with a client*, *you can hold them accountable*, *or ask whether they need help or assistance*. *If there are trusted people on groups*, *clients are more likely to do things*.*” (P6*, *woman*, *60-65y*, *nurse)*.

*Feedback and monitoring between professional and client*. An occasionally mentioned facilitator is feedback and monitoring between professionals and clients. Not only professionals monitor clients and hold them accountable, but also vice versa: *“Clients also indicate if social workers are not applying it and they can be very critical*, *most would say hey*, *but you have not washed your hands either*. *It is a good trigger to get everyone to implement it*.” *(P10*, *man*, *55-60y*, *manager)*.

*Professional role model towards client*. One participant reported that the lack of exemplary behaviour by professionals towards clients may negatively influence IPC: “*I think it starts with professionals themselves*, *and often a role model is lacking*.*” (P6*, *woman*, *60-65y*, *nurse)*.

#### Client interaction

*Peer influence between clients*. Some professionals reported that clients influence each other negatively. Imitation by clients of each other’s defiant behaviour is perceived as a barrier: *“They are all so impressionable*. *If one says I am not going to shower*, *ten others say the same*.*” (P6*, *woman*, *60-65y*, *nurse)*.

#### Organisational level

The majority of identified factors that could hamper or facilitate IPC were found at the organisational level.

*Organisational support and priority*. A frequently reported organisational barrier is the lack of structural organisational attention to IPC. Participants noted IPC only receives attention in case of an outbreak: *“IPC rarely receives attention*. *Only if practice forces us to pay attention to it*.*” (P5*, *man*, *35-40y*, *social worker)*. In line, participants perceived a lack of managerial support and priority as important barriers: *“There is no priority for IPC because the organisation does not see the need for it*.*” (P3*, *woman*, *35-40y*, *physician)*. Yet, participants perceived organisational awareness, including a sense of urgency, as an important facilitator of IPC.

*Educational system*. The most frequently discussed facilitator on the organisational level is structural education. Particularly education aimed at new employees, non-medically educated professionals, and clients is perceived as facilitating: *“Hygiene lessons are very interesting for clients with a mild intellectual disability to provide insight into the importance of hygiene*.… *Since IPC also depends on social workers*, *it is imperative to educate and train them*.*” (P9*, *woman*, *45-50y*, *supervisor)*. Nevertheless, a lack of education was often reported, and participants indicated the inclusion of IPC education in medical training courses aimed at nurses only: *“IPC education is not offered to all employees*. *It is included in the education and training for nurses*. *But not to other employees*.*” (P4*, *woman*, *40-45y*, *nurse)*.

*Time availability and staff capacity*. A strong reported barrier is high work pressure, which is often associated with staff shortages: “*Many colleagues are at home burned out*. *Only two of our team members are left*. *The work pressure is very high*, *we have so many tasks and they are all important*, *and IPC is not part of them*.*” (P6*, *woman*, *60-65y*, *nurse)*. In line, several participants indicated that high staff turnover may also hinder IPC: *“It is difficult to maintain a certain standard due to entry and exit of personnel*, *it is not feasible to explain every detail*.*” (P7*, *woman*, *60-65y*, *nurse)*.

*Task division and change coaches*. Participants often reported the presence of professionals in the organisation who are responsible for IPC coordination and implementation, such as an infection control professional or infection control committee, as facilitating: *“It is beneficial to hire an infection control professional*. *Now it is supplementary to the tasks employees already have*. *If someone has IPC as primary job*, *it will be emphasised more*.*” (P3*, *woman*, *35-40y*, *physician)*. Some reported a lack of professionals responsible for IPC, which is perceived as barrier: “*We do not have anyone responsible for IPC*, *while it is important for these themes to have someone who can advocate its importance and demand managerial support*.*” (P12*, *woman*, *35-40y*, *quality assurance officer)*. Several participants considered the presence of professionals who acts as driving forces for IPC as beneficial: *“One of our nurses has IPC as her area of ​​attention and focus*. *The fact that a few enthusiastic nurses are working on IPC is important and makes things easier*.*” (P10*, *man*, *55-60y*, *manager)*.

*Leadership and institutional policy*. Participants occasionally indicated a lack of IPC policy and insufficient involvement of management: “*Our management should provide more direction and guidance*. *Currently*, *they undertake little to no action*.*” (P3*, *woman*, *35-40y*, *physician)*. A lack of enforcement of non-adherence to IPC is also regarded as barrier: *“The organisation should emphasise the rules and regulations and state that some things are just not tolerated*. *Now*, *everyone can do whatever they want*, *without consequences*.*” (P4*, *woman*, *40-45y*, *nurse)*. Several participants perceived that organisational preparedness, i.e., clear measures in place in case of an outbreak, has a positive effect on IPC adherence since professionals know what is expected of them.

*Resources and materials*. The presence of adequate IPC materials/equipment and financial resources was considered important for IPC and perceived as hindering when these were lacking or inadequate: *“Resources*, *especially money*, *is the problem*.… *Some parts of the building lack soap dispensers and garbage bins*. *Also*, *recently our gloves were too large to wear*.*” (P10*, *man*, *55-60y*, *manager)*.

#### Community level

*Care sector-related social norms and culture*. Several participants indicated the domestic culture of disability care may lead to difficulties in IPC application: *“In disability care*, *you make a home while the culture in a hospital is one of high precision*. *It means that things which must be of high precision*, *such as hygiene or medication*, *requires a switch from employees from a relaxed atmosphere to a precise one*. *That makes it difficult*.” *(P8*, *woman*, *40-45y*, *supervisor)*. Correspondingly, mainly medically educated professionals noted that the sectoral shift from medical to behavioural aspects is not stimulating for IPC: *“There has been a sectoral shift from a nursing to a domestic culture*, *with focus on guidance*. *A shift is not always good*. *We switched from uniforms to regular clothes last year*. *The rule is to not take work clothes home*, *but people come and go in the same clothes*. *They think there are no risks*, *however*, *it is not a hygienic environment*: *so many people*, *so much complexity*.*” (P7*, *woman*, *60-65y*, *nurse)*. Yet, IPC is recognised as a collective concern and received more attention in disability care recent years: *“IPC has received a lot more attention recent years*, *also due to the introduction of various protocols regarding hygiene*, *which is positive*.*” (P9*, *woman*, *45-50y*, *supervisor)*.

*Interorganisational networks*. Collaboration between disability care facilities as well as collaboration with external health organisations (i.e., hospitals or public health services) is perceived as facilitating. Moreover, several participants indicated that sector-wide meetings have a positive influence on IPC: *“I think IPC is best performed in collaboration*. *A meeting is helpful since you can exchange ideas*. *It is a waste if every organisation has to invent the wheel themselves*. *We all deal with similar issues*.*” (P12*, *woman*, *35-40y*, *quality assurance officer)*. Despite the need for interorganisational collaboration, participants reported this is not very common: *“Collaboration between organisations is increasingly happening*, *but no common good in the sector*. *While working with other parties*, *you can strengthen each other*.*” (P1*, *woman*, *55-60y*, *manager)*.

#### Societal level

*Workforce*. Several participants reported the workforce shortage in care sectors as important barrier: *“The problem is our enormous staff shortages*. *They are happy someone is present*, *regardless of what their nails look like*. *Supervisors argue that if they are not here*, *no one is*. *Which is very sad*, *it does not benefit the quality at all*.” *(P3*, *woman*, *35-40y*, *physician)*.

*Involvement governmental agencies and cues to action*. An occasionally reported facilitator was sufficient information provision regarding IPC from governmental organisations: “*When I need information*, *I search the website of the national health institute*. *I also contact the public health service*, *with whom we have good contact*. *They are always very accessible and helpful in terms of protocols and information on what should be done*, *which works very well*.*” (P3*, *woman*, *35-40y*, *physician)*. In addition, few participants perceived a visit from the health inspectorate as facilitating, since these visits ensure IPC is addressed in the organisation: “*What also helps is a visit from the Healthcare Inspectorate*. *I wanted a separate employee for infection prevention*. *And when the inspectorate visited and acknowledged my idea*, *it facilitated the process*. *When it is advice from the inspectorate*, *the organisation has to do something*.*” (P1*, *woman*, *55-60y*, *manager)*. Other perceived facilitators are governmental initiatives directed to IPC, such as projects: *“The main reason for the introduction of an infection control committee was the introduction of the special chronic care project by the Ministry of Health*, *Welfare and Sport*.*” (P10*, *man*, *55-60y*, *manager)*.

### Professional-reported recommendations to improve IPC

Besides previously suggested recommendations (as described above) to improve IPC in disability care, such as the introduction of practical and tailored guidelines, and implementation of structural education and training among all professionals, participants reported several additional recommendations. Participants frequently recommended increasing client participation in IPC, e.g., by implementing hygiene lessons. *“I’d say involve the clients themselves*. *Something digital often appeals to them*. *They also enjoy hygiene lessons*.*” (P9*, *woman*, *45-50y*, *supervisor)*. Participants also occasionally suggested to include information provision and education on infections and IPC in the curriculum of social-agogic (e.g., social work) and nursing study programmes. Moreover, some recommended more guidance from management, while others suggested enforcement of non-adherence to IPC. Furthermore, participants highlighted the importance of including all facility staff, i.e., cleaning staff, kitchen staff and other support staff, when implementing IPC. A central need emerging among almost all professionals is the need for a tailored approach: *“IPC requires a different approach everywhere*, *due to the great diversity*. *It should be very tailor-made*, *depending on location and residents*.*” (P1*, *woman*, *55-60y*, *manager)*.

## Discussion

This study assessed perceived barriers and facilitators to IPC among professionals working in RCFs for people with IDD, for which we proposed an integrated theoretical framework. Our findings showed that factors influencing IPC can be categorised into the guideline, client, professional, professional interaction, professional client interaction, client interaction, organisational, community, and societal level. Our qualitative analysis revealed barriers and facilitators relating to various themes, with the following main themes: guidelines’ applicability to (work)setting, professionals’ cognitions and attitude (related to educational background), organisational support and priority, educational system, time availability and staff capacity, and task division and change coaches. An encompassing theme is the heterogeneous setting characterising disability care, indicated by the diversity in professionals, clients, and care needs. The results of the present study are in line with previous studies conducted in hospital and long-term care settings. One of our key findings is the influence of a professional’s cognitions and attitude on IPC implementation. This is supported by a survey study suggesting that efforts aimed at improving compliance with infection control practice in home care should focus on strategies to alter awareness, risk perceptions and other attitudinal factors [37]. Furthermore, a qualitative study among different healthcare workers (HCWs) indicated that the application of IPC heavily relies upon a shared belief in the importance of IPC, as well as proactivity and ownership of IPC practices [38]. Other key findings of the present study are the need for structural educational systems aimed at all professionals, and time and staff deficits as important barriers. This corroborates a recent Cochrane review that identified a need for training of all HCWs and a need for adequate staff numbers in IPC practices [39]. Another qualitative study examining barriers to IPC in nursing homes identified lack of knowledge and training, reliance on part-time staff, and high workload as important challenges [40]. Other studies on nurses’ compliance to IPC indicated the need for leadership, managerial support, and training in addition to addressing individual factors as awareness and attitude [41, 42]. While these studies are not specific to the disability care setting, there are commonalities across professional, organisational, and broader environmental factors that affect IPC.

Our findings also identified main professional-reported recommendations to improve IPC, including structural education and training of professionals, the introduction of tailored and practical IPC guidelines, and client participation. Previous reviews have emphasised the importance of patient engagement and education on IPC [43, 44]. Interventions including patient education were effective in improving patient’s knowledge and application of hand hygiene [45]. The implementation of IPC education systems for health professionals is also found to be effective [46, 47]. Moreover, in accordance with the present findings, previous studies indicated that introducing context-specific guidelines may facilitate successful IPC implementation, yet require coordinated actions at the organisational level [48]. Prior systematic reviews suggest the effectiveness of multifaceted interventions to promote IPC practice [49, 50].

### Strengths and limitations

The study is subjected to several strengths and limitations. A first strength is the exhaustive and integrative nature of the theoretical underpinnings of this study. By adopting an in-depth theoretical analysis of the facilitating and impeding factors for implementing change, the probability of developing a successful intervention is greater [18]. Yet, this study was both theory and data-driven, since inductive and deductive approaches for data analysis were adapted. Thereby, the study made sufficient use of existing theory but was not restricted by preconceived categories [51]. A second strength is the coding process, which was conducted by two trained researchers independently. In addition, data analysis was reviewed by a third researcher. Although facilities or institutions providing disability care may vary in and between countries, we presume our findings regarding barriers and facilitators might be similar throughout Western world countries. The integrated theoretical framework of this study is therefore expected to be applicable to other countries as well. A limitation of this study is the small sample size of only twelve participants. Nevertheless, data saturation [33] was reached. Another limitation is the use of convenience sampling instead of purposive sampling. Due to the sampling method, we could not report the exact response rate. We interviewed all participants who were willing to take part in the interviews. This could have led to some selection bias, as these participants might have been the most enthusiastic individuals. Nonetheless, we assume the sample was rather representative for the study population since we included professionals from several types of professions. Thirdly, we performed data analyses after all interviews had taken place. As a result, the findings from the first interviews did not guide the content of the following interviews. Another limitation is that facilitators and barriers at the client level were identified by professionals, and the client perspective was not obtained. An additional remark should be made regarding the descriptive manner of reporting barriers and facilitators. Since barriers and facilitators can occur simultaneously (e.g., attitudes can both be positive or negative) and are oftentimes associated with one another (e.g., cognitions like awareness are associated with attitude), the classification into being either a facilitator or barrier can overshadow the relevance of factors, their interplay, and possible interactions. Therefore, the relationships and interplay between factors should also be considered when interpreting our study findings.

### Implications for practice and research

IPC is important in disability care settings, which indicates the need for effective strategies to promote IPC. The COVID-19 pandemic magnified the recognition of the importance of IPC in care facilities, thereby assumably increasing support and commitment to implement and improve IPC, which heightens the relevance of our results. The perceived barriers and facilitators, and suggested professional-reported recommendations should be taken into account when developing future interventions. A first recommendation for practice would be to implement education and training specifically aimed at non-medically educated professionals. A second recommendation would be to involve clients in IPC education, which seems even more important in light of our findings that client interaction and professional client interaction are important interpersonal levels.

Our findings propose that strategies to promote IPC should target multiple factors (e.g., professionals’ attitude and support of board of directors and management) on multiple levels of influence (i.e., guideline, individuals, social environments, organisation, and the broader environment). A preliminary need emerged for improvement strategies aimed at the guideline, professional, and organisational level.

The present findings revealed regarding the social context next to the existing level ‘professional interaction’, known from extant theory [17, 22], two additional influencing social levels, namely ‘professional client interaction’ and ‘client interaction’. This provides new information for policymakers and intervention developers and enables them to install efforts targeting barriers and facilitators occurring on these specific social levels. Our findings moreover incorporate a ‘system approach’, by acknowledging the influence of broader community, i.e., disability care sector, (e.g., interorganisational networks and care sector-related social norms and culture) and societal factors (e.g., workforce and involvement governmental agencies). This yields extensive insights into all potential barriers and facilitators to IPC, which may again inform policy makers, intervention developers and researchers regarding efforts aimed at IPC improvement in disability care settings. The aforementioned seems extra relevant in light of previous studies indicating that social, organisational, and cultural factors influencing implementation behaviour are rarely considered when translating strategies into practice [52]. Our integrated theoretical framework conceptualises all these elements, which highlights the relevance of this framework for future research examining factors influencing IPC in disability care settings. To date the framework has not been validated, therefore, future studies are required to validate and refine the proposed integrated theoretical framework. In addition, future studies should explore the relationships between factors.

## Conclusion

As this study is the first in outlining perceived barriers and facilitators to IPC in a disability care setting, the findings can inform future practice improvements. The highest potential for improvements were identified at the guideline (e.g., applicability to setting), professional (e.g., cognitions and attitude towards IPC) and organisational level (e.g., organisational support and priority). Factors influencing IPC implementation are often multiple and interconnected. Strategies to promote IPC should be multifaceted and multilevel, and adopt a tailored approach (i.e., taking in mind the heterogeneous setting of disability care in terms of the diversity in professionals, clients, and care needs).

## Supporting information

S1 Appendix COREQ (COnsolidated criteria for REporting Qualitative research) checklist. (PDF)

S2 Appendix Interview topic guide. (PDF)

S3 Appendix Example of the coding process. (PDF)

## Abbreviations

IPC: Infection prevention and control
COREQ: COnsolidated criteria for REporting Qualitative research
HAI: Healthcare-associated infection
NH: Nursing home
LTCF: Long-term care facility
AMR: Antimicrobial resistance
RCF: Residential care facility
IDD: Intellectual and developmental disabilities
HCWs: Healthcare workers
