# Supplementary material for: Barriers and facilitators to infection prevention and control in Dutch residential care facilities for people with intellectual and developmental disabilities: A theory-informed qualitative study
Source: PLoS One. 2021 Oct 29;16(10):e0258701. doi: 10.1371/journal.pone.0258701 (PMC8555856; doi:10.1371/journal.pone.0258701)
Supplement: S2 Appendix — (PDF) [file pone.0258701.s002.pdf]

Additional file 2: Interview topic guide

**Background variables**	1. Could you introduce yourself?<br>o Age, gender, occupation, (work) experience.
	2. Could you tell me about the facility/institution you work at?<br>o Are there specific client groups you are in contact with?
**Influencing factors (i.e., barriers and facilitators)**	3. What is the first thing infection prevention and control (IPC) (in the disability care setting) reminds you of?
	4. What comes to mind when you think of infection prevention and control?<br>o To what extent do you consider IPC important?<br>o What role did IPC play during your education/studies?<br>o Does your facility/institution offer IPC education/are you taking IPC courses or training?<br>o Are you aware/familiar with IPC guidelines (e.g., hygiene protocols) in your institution?<br>i. To what extent do these guidelines play a role in your work? (Hand hygiene, personal protective equipment, clothing regulations, hygienic working environment, resources and materials)<br>ii. Are these guidelines adequate? (e.g., comprehensibility, practicality)<br>o How do you acquire IPC information?
	5. To what extent does IPC play a role at work?<br>o What do you think your colleagues think of (the importance of) IPC?<br>o To what extent do people check/correct each other on IPC/hygiene?<br>o Do you make a distinction regarding IPC between different client groups (based on care needs)?<br>o To what extent do you stimulate/motivate clients to apply hygiene/IPC measures?<br>o Could you share some experiences with IPC (measures)?
	6. To what extent does IPC play a role on an organisational

	level? (e.g., policy, formal agreements).<br>o How much attention is paid to IPC?<br>o Is enough priority given to IPC?<br>o Who deals with/are involved in IPC/hygiene in your organisation?
**Recommendations**	7. Do you think there are changes needed with regards to IPC in your organisation? And if so, what would you recommend?
	8. What needs to change so you can focus more on IPC?
Abbreviation: IPC infection prevention and control	
