# Supplementary material for: Barriers and facilitators to infection prevention and control in Dutch residential care facilities for people with intellectual and developmental disabilities: A theory-informed qualitative study
Source: PLoS One. 2021 Oct 29;16(10):e0258701. doi: 10.1371/journal.pone.0258701 (PMC8555856; doi:10.1371/journal.pone.0258701)
Supplement: S3 Appendix — (PDF) [file pone.0258701.s003.pdf]

Additional file 3: Example of the coding process

Participant	Quote	Code	Theme	Level
Participant #4 (woman, 44y, nurse).	"IPC has to do with mentality. If someone cannot be bothered and does not give it enough effort, you will not get there. It is often an issue of laziness".	Negative professional attitude	Professional's cognitions and attitude	Professional
Abbreviation: IPC infection prevention and control				
